# Implementation Strategies: Bridging Obstetrics and Long-Term Care

**DOI:** 10.1016/j.ajogmf.2026.101998

**Published:** 2026-08-01

**Authors:** S. Michelle Ogunwole, Sumaiya Adam, Graeme N Smith

**Affiliations:** 1 Department of Medicine, Johns Hopkins School of Medicine, United States of America; 2 Department of Obstetrics and Gynaecology, University of Pretoria, Pretoria, South Africa; 3 Department of Obstetrics and Gynecology, Queen’s University, Kingston, Canada

**Keywords:** postpartum, cardiovascular risk, cardiovascular disease, screening, lifestyle modification

## Abstract

It is well recognized that certain adverse pregnancy outcomes are associated with underlying cardiovascular risk factors identifying individuals who are at greater long-term risk of cardiovascular disease. As such, it is recommended that cardiovascular risk screening, counselling and lifestyle modification occurs within the first year postpartum for health preservation, disease prevention, and future pregnancy planning. Obstetrical care providers are ideally situated to identify these individuals and ensure that a follow-up plan, understood by the patient and the care providers, is put in place. This invited review focuses on the postpartum screening expectations including implementation strategies that bridge obstetrical and longer-term care.

## Introduction

It is well established that certain adverse pregnancy outcomes (APOs) (e.g. hypertensive disorders of pregnancy (HDP), gestational diabetes (GDM), idiopathic preterm birth, delivering a growth restricted baby, placental abruption leading to delivery, stillbirth),^1,2^ are associated with an increased risk of future cardiovascular disease (CVD) and can reliably identify postpartum individuals who have underlying, often unrecognized, cardiovascular disease risk factors (CVR).^2–4^ The data to support this has been well laid out in this AJOG MFM Virtual Special Issue. Women with a known CVR prior to pregnancy (e.g. chronic hypertension, diabetes, hypercholesterolemia) should already be counselled and closely followed for CVD risk reduction. The development of one or more of these APOs is a way of identifying women, who are not already involved in a CVD risk-reduction health plan, who would benefit from screening, counselling and close clinical follow-up. Importantly, pre-pregnancy CVR, APOs, and subsequent CVD risk are not equitably distributed across populations, reflecting longstanding structural inequities in health and health care access. Individuals from racially minoritized and socioeconomically marginalized groups experience higher burdens of APOs and cardiometabolic risk factors and face greater barriers to postpartum follow-up and prevention—inequities that contribute to persistent disparities in maternal and cardiovascular mortality.^5–7^ Therefore, the postpartum period provides an opportunity for health preservation and disease prevention, future pregnancy planning, and, if equitably implemented, an opportunity to reduce disparities in long-term cardiovascular outcomes across populations.^8^

## Postpartum Follow-up

Following APOs associated with future CVD, numerous retrospective and prospective cohort and database studies, conducted months to years postpartum, have shown that these individuals are more likely to exhibit traditional CVR upon screening. In fact, 17% of these individuals, within the first year postpartum, met the criteria for the metabolic syndrome and 50%, based on detectable risk factors, had an increased calculated lifetime risk of CVD when compared to individuals who experienced uncomplicated pregnancies.^9^ It is estimated that at least 20% of all pregnant women will have one or more of these APOs and would potentially benefit from postpartum follow-up beyond the traditional six weeks.^10^ As previously recommended (Table 1), screening should be done within the first year postpartum.

**Figure 1.**
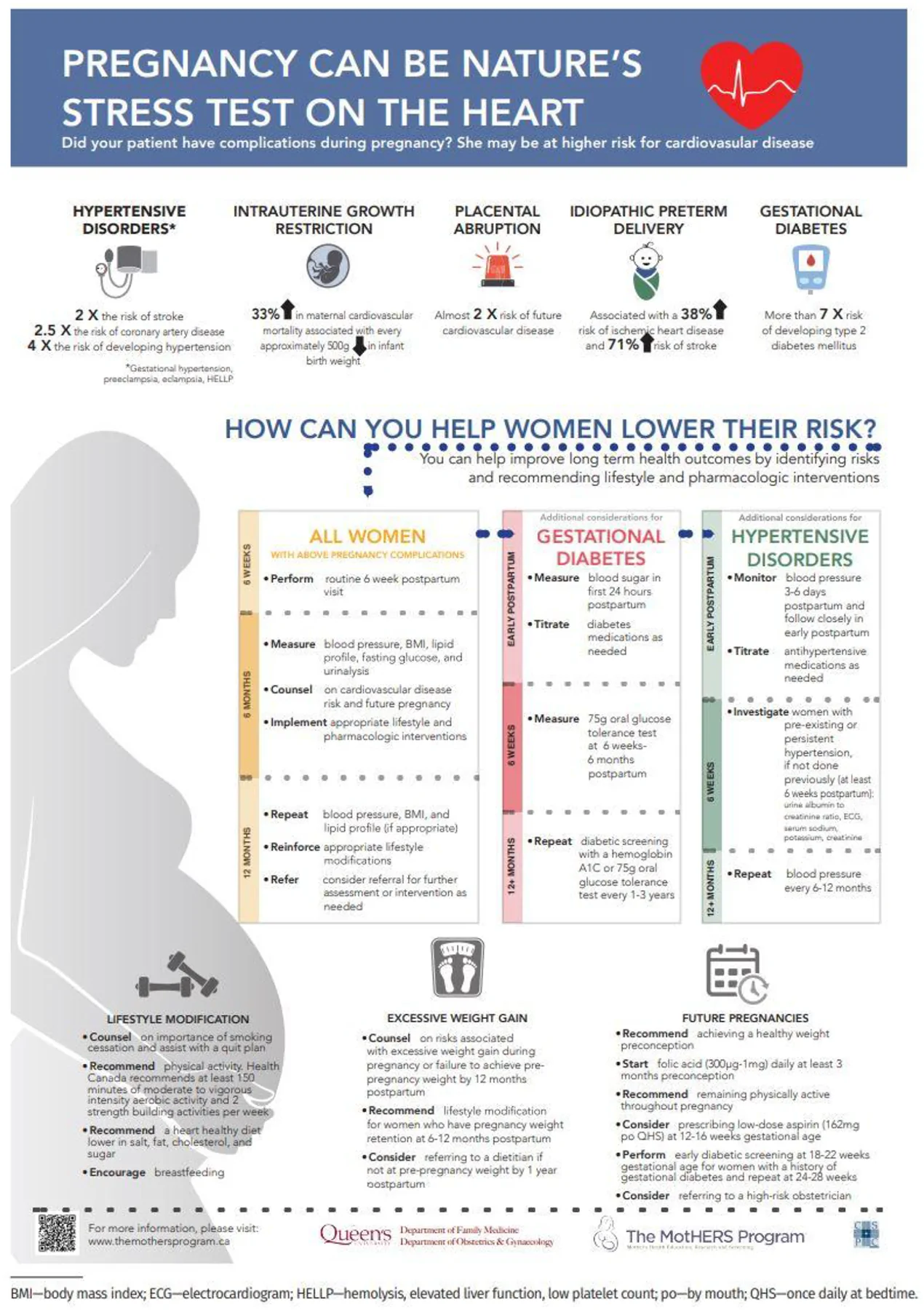
Reference tool for primary care providers on management of adverse pregnancy outcomes in the postpartum period. (Used with permission *Canadian Family Physician*^8^)

**Figure 2.**
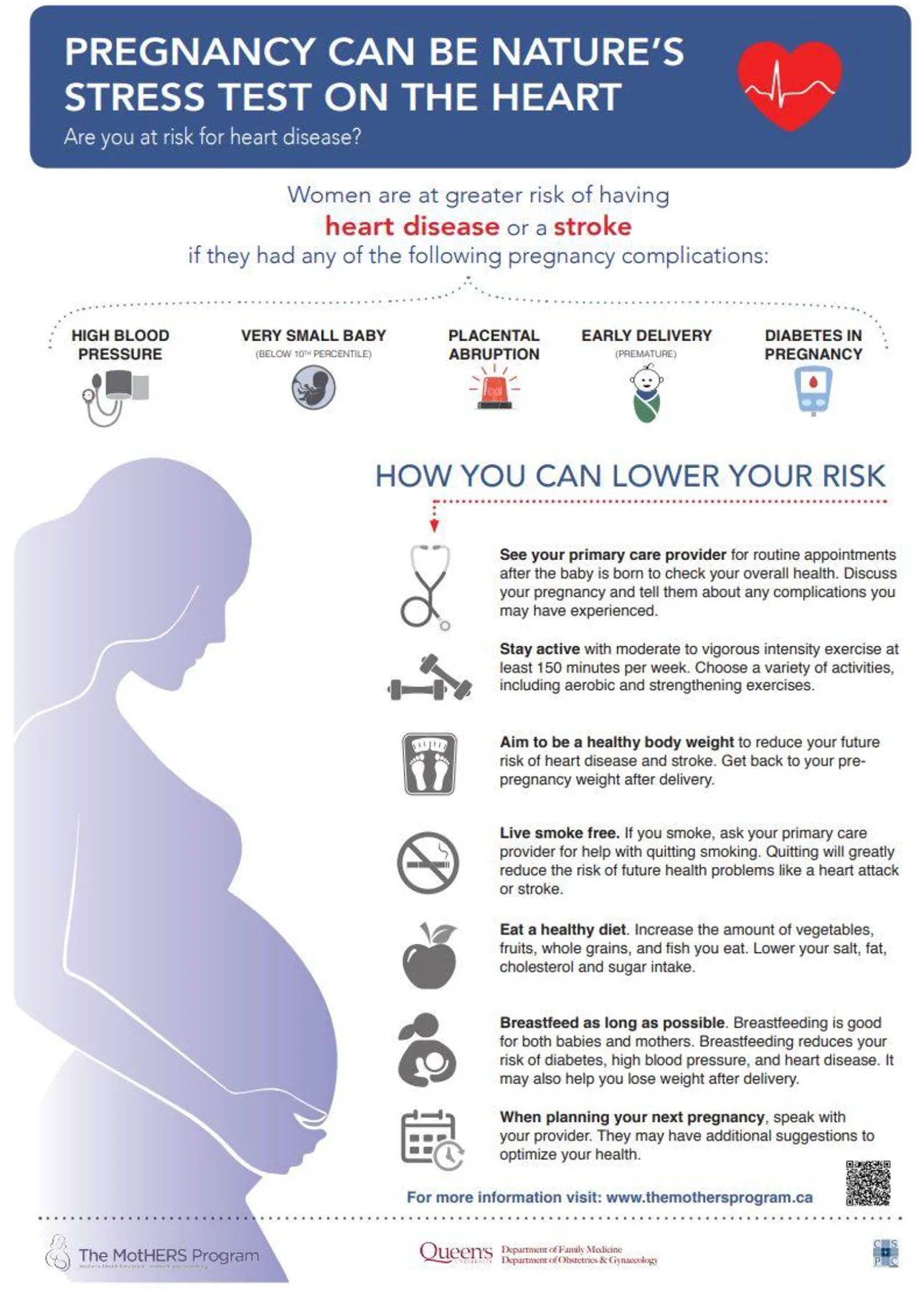
Poster tool for patients to increase awareness of adverse pregnancy outcomes and the association with cardiovascular risks and future cardiovascular disease. (Use with permission *Canadian Family Physician*^8^)

Unfortunately, follow-up of women postpartum, especially those who have had APOs, can be fragmented with many health care providers being unclear on the associated risks and what their roles or responsibilities might be for screening and longer-term follow-up and management.^11–13^ Along that lines, a reproducible reference tool for primary care providers on the postpartum screening and management following an APO is provided (Figure 1). Women’s own risk perception of future CVD following an APO tends to be low.^14^ A reproducible tool to provide to affected women, to increase awareness of APOs and the association with CVR and future CVD, is provided (Figure 2). This critical transition from obstetrical-focused care to longer-term primary health care requires coordination, structure, and better knowledge mobilization for both the patients and the health care providers.

Several barriers to postpartum screening have been identified. From the patient perspective, these include limited perception of cardiovascular disease risk, poor health literacy with underestimation of personal risk, fear-driven avoidance, time constraints, inconvenience, and financial costs, while system-level barriers include the absence of clear guidelines and fragmented, unstandardized postpartum care..^15,16^ However, a family history of CVR or CVD, personal health advocacy, strong family support, and positive experience with the health care system were identified as factors that facilitated an individual’s accessing follow-up and screening.^15,16^ Despite these potential barriers, a review of postpartum CVR management has called for the development of structured postpartum CVR screening programs after complicated pregnancies.^17,18^ Close collaboration with primary health care and use of telemedicine, especially for those who live rurally or remotely, should be considered. While postpartum CVR screening clinics do exist,^10^ primarily in Canada and the United States, they are typically based in tertiary care centres and have limited capacity. CVR screening, in the general population, is usually managed by primary care providers; ideally postpartum risk screening should follow a similar model with the support of a multidisciplinary team. The obstetrical care provider is ideally situated to identify the individuals with these APOs and should ensure that a plan for follow-up is put in place and that the patient is counselled accordingly and understands the reasons and recommendations. Later, preconceptual planning visits with the obstetrical care provider to discuss future pregnancy risk reduction would be a reasonable expectation. It should be the role of the primary health care providers (e.g. family physicians, general internists, nurse practitioners) but also specialists (e.g. gynecologists, cardiologists) to do the appropriate physical exam and biochemical testing and provide any longer-term medical follow-up and/or clinical referral.^19^

Health system–based interventions that focus on transitions of care in the postpartum period, prioritize interrelated health and social needs of individuals at risk of readmission and disease progression, improve patient awareness, and offer postpartum screening for cardiovascular risk factors are the crux of postpartum care.^20^

## Postpartum cardiovascular screening practices

In 2010, with a knowledge translation (KT) grant, the Maternal Health Clinic (MHC),^10^ as part of The MotHERS Program^™^ (www.themothersprogram.ca) was started. This followed the publication of the *PreEclampsia New Emerging Team* (PE-NET) prospective cohort.^21^ The development of CVD requires the exposure to CVRs for years, if not decades, thus, the PE-NET cohort aimed to determine at what point postpartum would individuals with preeclampsia be found to have CVRs. Women with preeclampsia or an uncomplicated pregnancy underwent CVR screening at year one, three and five postpartum. Within the first year, those women who had had a pregnancy complicated by preeclampsia were already more likely to have CVRs.^21^ This included a threefold greater prevalence of the metabolic syndrome^22^ and higher CVD risk scores.^23^ There was no significant change in CVR prevalence in the two groups beyond the first year, identifying that screening should not be delayed (Table 1). Maternal Health Clinic data from the heterogenous group of postpartum women following all the associated APOs, was comparable to that of the PE-NET preeclamptic group alone.^9^ By the time the KT grant expired, postpartum CVR screening had become standard of care in Kingston, Canada; the clinic has now been in operation for fifteen years. Unfortunately, despite universal health care in Canada, not all individuals referred to the MHC attend.^16^ In fact, those that do not attend tend to be a higher risk group with a greater prevalence of obesity, young age, smoking, and lower socioeconomic status. Despite attempts to improve the uptake by this group, including offering virtual visits and the proposal to move clinics out of the hospital, the uptake remained unchanged.^24^ Many similar postpartum maternal CVR screening clinics have been initiated across Canada and the USA.^25^ Many other examples of postpartum CVR screening clinics exist, including the nurse practitioner-led cardiovascular prevention clinic in Australia.^26^ One particularly successful clinic, with greater than 94% follow-up, is the family physician run *Birth-Related Cardiovascular Health* (BIRCH) clinic in rural British Columbia, Canada (Birth-Related Cardiovascular Health (BiRCH) Clinic - Health Quality BC).^27^ It’s high postpartum follow-up rate is likely related to including a well-baby six-month postpartum visit at the same time as the maternal CVR screening and counselling.

The ideal location of follow-up has not been determined; many individuals, even in high-income countries, lack health insurance that would cover postpartum risk screening, and many others lack a primary health care provider. Strengthening transitional care, integrating structured screening into postpartum guidelines, expanding insurance coverage, digital health innovations, and enhancing provider training are essential to close clinical gaps and improve long-term cardiovascular outcomes.^15^ The International Federation of Gynecology and Obstetrics (FIGO) recommends that all women, following an APO, should be provided with the FIGO Pregnancy Passport^27,28^ to empower the women themselves to take ownership of their follow-up. (See article by Wallen et al., *FIGO Pregnancy Passport and Beyond: Strengthening Continuity of Maternal Postpartum Follow-Up and Caring for Future Long-term Health* in this AJOG MFM virtual special issue).

## Specific follow-up recommendations

- Hypertensive disorders of pregnancy: Blood pressure (BP) should be followed closely postpartum, especially in those women discharged home on anti-hypertensive medication. Close home BP recording with physician monitoring was shown to result in better BP control at six months^29^ and three years^30^ postpartum compared to usual care. Further, improvement in echocardiographic measurements^31^ suggests that early and tighter control may improve cardiac remodelling postpartum which may reduce the longer-term risks of CVD.
- Gestational Diabetes: Recognizing that approximately 30% of these women will go on to develop type II diabetes, most international societies recommend an oral glucose tolerance test (OGTT) (75 g) between 4 weeks and 6 months postpartum. If the OGTT is normal, it is recommended they have a fasting glucose and/or a HbA1c every 1–2 years for screening purposes.^32^ Women should also be encouraged and supported to breastfeed for at least four months to reduce the risk of neonatal hypoglycemia, childhood obesity, and later onset diabetes in both the mother and child.
- All APOs.
  - Blood pressure should be checked at 3–6 months postpartum then once to twice yearly. For ease of remembering and facilitating follow up, this could be done in conjunction with standard early child clinical assessments/vaccinations.
  - Weight, height, waist circumference and BMI calculation at 3–6 months; repeat yearly and prior to any future pregnancy to assess return to previous pregnancy BMI and/or weight loss in obese women. Lifestyle intervention between 6–12 months improves weight loss and waist circumference.^33^
  - Lipid profile (+/− lipoprotein (a) depending on national guidelines) at 3–6 months or later if still breastfeeding. If abnormal, repeat after a trial of lifestyle modification (6–12 months). If normal, repeat every 2–3 years or as per national guidelines.
  - Serum creatinine/eGFR and urine protein quantification at 3–6 months. Recognizing the longer-term risk of kidney dysfunction following a HDP,^34^ persistent urine proteinuria following a HDP may benefit from referral and follow-up with nephrology.
  - Edinburgh Postnatal Depression Scale (or other recommended depression/anxiety screening tool) at each clinical visit in the first year postpartum.

## Lifestyle Modification recommendations

Structured postpartum lifestyle intervention programs have been shown to improve markers of cardiovascular health as well as physical fitness.^35^ An Australian randomized trial of different lifestyle intervention strategies, from 6 to 12 months after an HDP, found that intervention groups had improved weight loss and waist circumference compared to usual care;^33^ more extensive lifestyle intervention (education intervention (information package, physician-led postpartum clinic, dietician consultation) and a telephone-based programme) was not better than the education intervention alone. Along the same lines, women who showed up to the MHC and had CVR screening and basic lifestyle counselling, demonstrated improvements in interpregnancy weight reduction.^36^

The Diabetes Prevention Program (DPP) was a landmark U.S.-based, multicenter randomized controlled trial demonstrating that intensive lifestyle intervention and metformin reduce progression from prediabetes to type 2 diabetes.^37^ In a secondary analysis comparing women with and without a prior history of GDM, both intensive lifestyle intervention and metformin reduced incident type 2 diabetes by approximately 50% over three years among those with prior GDM with persistent benefit observed over 10 years of follow-up in the DPP Outcomes Study.^38^ Consistent with this evidence, the American Diabetes Association (ADA) recommends intensive lifestyle intervention and consideration of metformin for diabetes prevention among individuals with prior gestational diabetes^39^. Similarly, the American Heart Association recommends referral to intensive lifestyle interventions for women with gestational diabetes, and other APOs;^40^ benefit of metformin following HDP has not been established.

Interventions to improve short-term and long-term CVD risks, such as the American Heart Associations (AHA) *Life’s Essential 8* have real potential, particularly in the first year postpartum, by offering a practical, evidence-based approach to CVD prevention, particularly for individuals with a history of APOs. It encompasses eight modifiable domains (i.e. diet, physical activity, smoking cessation, sleep, body weight, lipids, blood glucose, blood pressure), many of which may worsen or newly emerge after complicated pregnancies. The postpartum transition provides an important opportunity to reassess these factors and support continuity from obstetric to primary care. *Life’s Essential 8* allows for individualized goal setting that accounts for patient priorities and social context, making it a flexible and equitable tool for postpartum CVR reduction.^20^

## Exercise

Exercise in the postpartum period plays a critical role in reducing long-term CVR, particularly for patients who have experienced APOs. Physical activity improves blood pressure, insulin sensitivity, lipid profiles, and body composition, all of which are key to reduce or prevent CVR that may emerge months to years after delivery.^41–43^

Postpartum exercise does not need to be intensive to be beneficial. The World Health Organization^44^ emphasizes the principle that “*Every Move Counts*,” highlighting that any amount of physical activity is better than none. For postpartum individuals, particularly those balancing recovery, childcare, and work demands, incorporating movement into daily routines, such as walking, light resistance training, or active play with an infant, can be both realistic and effective. Even short bouts of activity accumulated throughout the day contribute meaningfully to cardiovascular health.

Importantly, exercise prescriptions should be individualized, taking into account a woman’s obstetric history, mode of delivery, recovery status, and their personal preferences. Ideally, postpartum women should be encouraged to progressively increase to 120–150 min/week of moderate-to-vigorous physical activity (e.g. brisk walking) including muscle/resistance strength exercises.^45^ When appropriately supported by healthcare providers, regular physical activity in the postpartum period represents a low-cost, accessible, and powerful strategy to mitigate CVR in individuals with a history of APOs.^45^

## Diet

Dietary interventions in the postpartum period are an important strategy for reducing long-term CVR. Nutritional patterns such as the Mediterranean and *Dietary approach to Stop Hypertension* (DASH) diets have been consistently associated with improvements in blood pressure, lipid profiles, insulin sensitivity, and overall cardiovascular health.^46^ The Mediterranean Diet emphasizes fruits, vegetables, whole grains, legumes, healthy fats, and lean proteins, while the DASH Diet focuses on sodium reduction alongside increased intake of potassium-, calcium-, and magnesium-rich foods.^47^ However, effective postpartum dietary counseling must extend beyond prescriptive diet models to consider local food environments, cultural preferences, affordability, and food security.^48^ Social determinants of health, including income, housing stability, access to grocery stores, and time constraints, strongly influence dietary choices in the postpartum period. Rigid adherence to named dietary patterns may be unrealistic or inequitable for many patients.

Adapting heart-healthy dietary principles to locally available and culturally familiar foods can improve feasibility and sustainability. Simple strategies such as increasing vegetable and fiber intake, reducing highly processed foods, and reducing excess sodium and added sugars can confer cardiovascular benefits without increasing financial burden. Integrating nutrition counseling with social support services is essential to addressing structural barriers and promoting equitable CVR reduction after APOs.

## Benefits of breastfeeding

Breastfeeding in the postpartum period has been associated with meaningful cardiovascular benefits.^49,50^ Lactation is linked to improved glucose metabolism, more favorable lipid profiles, lower blood pressure, and enhanced postpartum weight regulation—factors that are particularly relevant given the elevated long-term CVR observed after APOs. Longer duration of breastfeeding has been associated with a lower incidence of hypertension, type 2 diabetes, and CVD later in life.^51^

From a mechanistic perspective, it is thought that breastfeeding promotes metabolic resetting after pregnancy and may mitigate persistent cardiometabolic changes associated with complications such as HDP or GDM.^52^ The World Health Organization recommends exclusive breastfeeding for the first six months when possible, recognizing both maternal and infant health benefits.^53^ Importantly, breastfeeding should be encouraged without pressure or stigma. Structural barriers, including workplace policies, access to lactation support, and social determinants of health, must be addressed to ensure equitable opportunity for those who choose to breastfeed as part of postpartum CVR reduction.^54^

## Future pregnancy counselling

Postpartum screening and counselling following an APO should also include future pregnancy planning; different health care providers may be involved in the different aspects of postpartum care and counselling.

Folic acid (400ug) should ideally be initiated three months prior to conception to reduce the risk of fetal neural tube defects. Certain medical conditions (e.g. diabetes, a history epilepsy, obesity, etc.) may all benefit from taking a higher dose (≥1mg) of folic acid. While many women will obtain a small or moderate amount of folic acid in their diets (e.g. dark leafy greens, legumes, fortified grains), most will require supplementation. Prenatal vitamins often include 1mg of folic acid which, for most women, is adequate.

Iron deficiency/iron deficiency anemia is very prevalent in women of reproductive age and can have significant impacts on the pregnancy and fetal/neonatal neurodevelopmental outcomes. Preconception dietary iron intake and/or iron supplementation should be discussed. Early testing (ferritin) and treatment with oral iron supplementation and/or iv iron should be considered if ferritin levels are <50ug/L.^55^

There is an increasing prevalence of overweight, obesity or severe obesity in women of reproductive age.^56^ For a significant number of women attending an obesity clinic, excessive gestational weight gain—along with postpartum weight retention and/or additional postpartum weight gain—was identified as a key trigger for long-term weight gain and the development of obesity.^57^ Postpartum weight retention and/or weight gain, even by as little as three BMI units compared to previous prepregnancy, is associated with an increase risk of APOs.^58^ Therefore, it should be recommended to all postpartum women to delay a future pregnancy until at least their previous preconception BMI has been attained. As weight loss is so important for future pregnancy and longer-term health including CVR reduction, a multidisciplinary approach involving allied health professionals (e.g. dietician, social worker, physical therapist, personal trainer, psychologist, etc.) may play a key role in successful behavioral and/or lifestyle modification. Interventions in the form of dietary counselling and follow-up, structured physical activity and creating realistic objective targets and timelines are the most effective intervention strategies for postpartum weight loss.^59^ Despite evidence of increasing use of GLP-1 receptor agonists to facilitate postpartum weight loss,^60^ there is very limited evidence to support this practice, including safety with breastfeeding, and no data on longer-term outcomes in this patient population including impact on CVR and CVD. However, this is an area of active investigation; for example, the Semaglutide for the Treatment of Glucose Intolerance in Women with Prior Gestational Diabetes (SERENA) trial is an ongoing randomized controlled trial evaluating whether Semaglutide reduces progression to type 2 diabetes among women with prediabetes following a GDM-affected pregnancy (https://clinicaltrials.gov/study/NCT05569772).

In women at increased risk of early onset preeclampsia, the use of low dose aspirin (LD-ASA)^61^ is an effective risk-reduction strategy supported by many international societies.^62,63^ The LD-ASA needs to be started prior to 16 weeks gestation to be most effective. In those women at high risk for fetal growth restriction and/or preterm birth, initiation of LD-ASA prior to 16 weeks has also been recommended.^64^ For those women who had a previous pregnancy complicated by gestational diabetes, earlier gestational glucose screening is recommended.

## Conclusion

Up to 94% of population-attributable CVRs in women are modifiable.^65^ Therefore, CVD is potentially preventable with screening, risk identification, lifestyle modification, and therapeutic intervention. Implementing postpartum CVR screening and intervention after APOs could provide substantial long-term health and survival benefits for women years before menopause, when CVR screening is routinely recommended by international societies.^66^

## Figures and Tables

**Table 1.** Rationale for Cardiovascular Risk Screening (CVR) within first year postpartum^19^

CVR are already identifiable within the first year for the majority of women who are found on further follow-up to have underlying risk factors
Delaying intervention for those who have underlying CVR potentially allows the disease process to progress and become more established, which may make it less likely to be reversible
Given that these CVRs, including weight retention or weight gain postpartum, increase the risk of future pregnancy complications, there is an opportunity for intervention to potentially improve future pregnancy outcomes. Therefore, earlier identification allows for more realistic timeline goals before a subsequent pregnancy
Delaying long-term follow-up increases the chance of loss to follow-up over time. Therefore, follow-up within a relatively short time postpartum, but allowing for the normal physiologic changes of pregnancy to resolve, will likely lead to a larger number of eligible women being screened
Guidelines for women who had a pregnancy complicated by gestational diabetes already recommend an oral glucose tolerance test between 6 weeks and 6 months postpartum
